# Tumour Immunotherapy and Applications of Immunological Products: A Review of Literature

**DOI:** 10.1155/2024/8481761

**Published:** 2024-10-24

**Authors:** Angus Nnamdi Oli, Samson Adedeji Adejumo, Adekunle Babajide Rowaiye, Joyce Oloaigbe Ogidigo, Jarrad Hampton-Marcell, Gordon C. Ibeanu

**Affiliations:** ^1^Department of Pharmaceutical Microbiology and Biotechnology, Faculty of Pharmaceutical Sciences, Nnamdi Azikiwe University, Awka 420211, Nigeria; ^2^Department of Biological Sciences, University of Illinois, Chicago, 845 West Taylor, Chicago 60607, Illinois, USA; ^3^Department of Pharmaceutical Microbiology and Biotechnology, Faculty of Pharmaceutical Sciences, Federal University Oye Ekiti, Oye, Ekiti State, Nigeria; ^4^National Biotechnology Development Agency, Abuja 900211, Nigeria; ^5^Department of Pharmaceutical Science, North Carolina Central University, Durham 27707, North Carolina, USA

**Keywords:** biologics, cancer, immune therapy, immunological products, malignant, tumors

## Abstract

Malignant tumors, characterized by uncontrolled cell proliferation, are a leading global health challenge, responsible for over 9.7 million deaths in 2022, with new cases expected to rise to 35 million annually by 2050. Immunotherapy is preferred to other cancer therapies, offering precise targeting of malignant cells while simultaneously strengthening the immune system's complex responses. Advances in this novel field of science have been closely linked to a deeper knowledge of tumor biology, particularly the intricate interplay between tumor cells, the immune system, and the tumor microenvironment (TME), which are central to cancer progression and immune evasion. This review offers a comprehensive analysis of the molecular mechanisms that govern these interactions, emphasizing their critical role in the development of effective immunotherapeutic products. We critically evaluate the current immunotherapy approaches, including cancer vaccines, adoptive T cell therapies, and cytokine-based treatments, highlighting their efficacy and safety. We also explore the latest advancements in combination therapies, which synergistically integrate multiple immunotherapeutic strategies to overcome resistance and enhance therapeutic outcomes. This review offers key insights into the future of cancer immunotherapy with a focus on advancing more effective and personalized treatment strategies.

## 1. Introduction

A tumor is a mass of tissue caused by the aberrant and excessive proliferation of cells. Compared with the normal surrounding tissue, tumors continue to grow in an uncoordinated manner even after the original trigger is removed [1]. Tumors could either be benign when they do not invade nearby tissue or metastasize throughout the body; premalignant when they have not spread but have the potential to become malignant; or malignant when they are cancerous [2]. Malignant tumors, also referred to as cancers are group of diseases characterized by their unregulated cellular proliferation, their capacity to infiltrate adjacent tissues, and their potential to metastasize to distant regions of the body [3]. These tumors pose a significant health burden globally, being one of the leading causes of death. Cancer is a leading cause of mortality globally, with nearly 20 million new cases and approximately 9.7 million cancer-related deaths reported in 2022 alone. The global burden of cancer is projected to increase significantly in the coming years, with predictions estimating that the incidence of newly diagnosed cancer cases could reach 35 million annually by 2050, primarily due to population growth and aging. This increase underscores the importance of ongoing efforts in cancer prevention, early detection, and treatment, including advancements in immunotherapy and other innovative therapeutic strategies. Traditional cancer treatments, including surgery, radiation therapy, and chemotherapy, have served as the backbone of oncological care for decades. While these approaches can be effective, particularly in early-stage cancers, they often come with significant limitations, including toxicity, resistance, and recurrence [4]. Additionally, these treatments do not always adequately address the underlying mechanisms of tumor immune evasion, which plays an important role in cancer progression [5].

Tumor immunotherapy, also known as cancer immunotherapy, harnesses the body's immune system to recognize and destroy malignant cells, representing a groundbreaking strategy in oncology. Immunotherapy targets the dynamic interactions between the tumor cells and immune system, offering the potential for more durable responses and fewer side effects [5]. Through numerous experiments and clinical studies, immunotherapy has shown significant advantages over conventional antitumor therapies particularly in prolonging progression-free survival and overall survival [6]. The tumor microenvironment (TME) is crucial in tumorigenesis, as nonmalignant cells in the TME accelerate uncontrolled cell proliferation [7]. Understanding the complex interactions between tumor cells, the TME, and the immune system is critical for designing effective prophylactic and therapeutic interventions against cancer [8]. Despite the promise of immunotherapy, challenges remain in its broader application. For instance, not all patients respond to immunotherapy, resistance can develop over time, and there are immune-related adverse events (irAEs), which require careful management [9]. Understanding the mechanisms of resistance and developing strategies to overcome these barriers are critical areas of ongoing research. This review discusses recent breakthroughs with tumor immunotherapy, molecular and cellular mechanisms of immune cells towards preventing and treating tumor cells as well as limitations and challenges associated with tumor immunotherapy. We also seek to highlight potential opportunities and future directions in tumor immunotherapy.

## 2. Tumor and Cancer Therapies

Cancer treatment has evolved through multiple modalities, each offering unique advantages and limitations.  Table 1 provides a summary of the major types of cancer therapies, highlighting their mechanisms, pros, and cons.

### 2.1. Tumor Cells and Their Antigens

**Figure 1:**
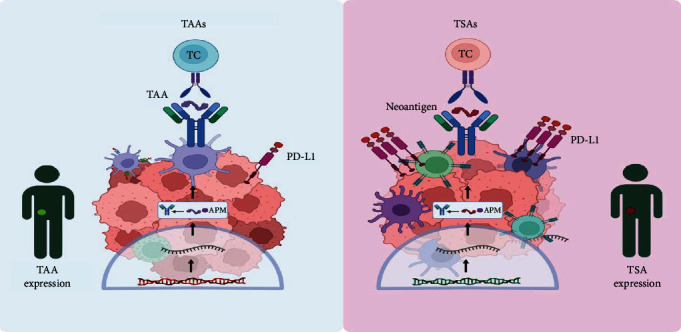
Tumor antigen types. TAAs immune response (left) and TSA immune response (right). APM, antigen-presenting machinery; PD-L1, programed death-ligand 1; TAAs, tumor-associated antigens; TSA, tumor-specific antigen.

Tumor cells differ significantly from normal cells due to genetic mutations and epigenetic alterations that result in abnormal protein expression. These proteins, known as tumor antigens, are important in tumorigenesis and serve as targets for immune recognition and therapeutic intervention. Tumor antigens can be broadly classified into two distinct groups: tumor-associated antigens (TAAs) and tumor-specific antigens (TSA) as shown in Figure 1 [26].

#### 2.1.1. TSAs

TSAs are small proteins (polypeptides) expressed only on tumor cells and are generated from abnormal or nonexpressed proteins in normal cells. These antigens typically arise from mutated proteins due to genetic alterations in the tumor. Examples include aberrant rat sarcoma (Ras) and *p53* gene products [27]. Neoantigens, which are a subset of TSAs, are produced by mutations in the tumor genome that lead to the generation of novel peptides. For instance, mutations in the Kirsten rat sarcoma virus (*KRAS*) gene in pancreatic cancer can result in the formation of neoantigens capable of being identified by the immune system, serving as unique markers that can be targeted by personalized cancer treatments [28].

#### 2.1.2. TAAs

TAAs are overexpressed or aberrantly expressed normal proteins that can be found in both tumor and normal tissues, though they are typically present at much higher levels in tumors. These antigens may not structurally differ from normal cellular antigens, which can sometimes limit their specificity as therapeutic targets. They can be used as diagnostic markers, such as *α*-fetoprotein and carcinoembryonic antigen [29]. TAAs trigger T lymphocyte responses via the major histocompatibility complex (MHC) and can lead to the destruction of tumor cells through cytotoxicity mediated by CD8 cells and antibody-dependent complement activation [8, 30]. Human epidermal growth factor receptor 2 (HER2) is a well-known TAA overexpressed in certain breast cancers. HER2-targeted therapies, such as trastuzumab, have significant efficacy in treating HER2-positive breast cancer by targeting this overexpressed protein [31]. The identification and targeting of these tumor antigens are key to the development of effective cancer immunotherapies.

## 3. Mechanisms of Immune Responses to Tumors

The immune system is essential for identifying and eradicating tumor cells through a complex network of cellular mechanisms. These mechanisms involve various immune cells, signaling pathways, and molecular interactions that collectively orchestrate the antitumor immune response [32].

### 3.1. Key Immune Cells in Antitumor Responses

**Figure 2:**
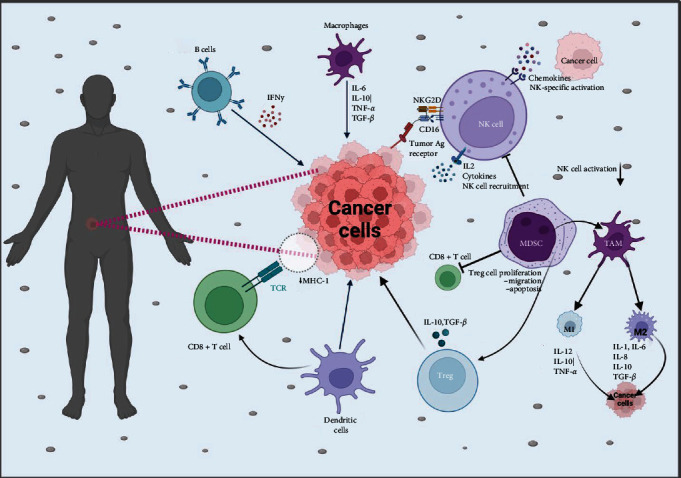
Immune cells involved in tumor cells response. CD8^+^ T cells: recognize and destroy tumor cells. NK cells: perforating and lysing of tumor cells. NKT cells: tumor cells death and release of cytokines. Macrophages: support inflammation that promotes tumor growth. DCs: priming the T cells to respond against a tumor. Tregs: inhibit antitumor immune response. MDSCs suppress the antitumor function of the NK and T cells. DCs, dendritic cells; IFNy, interferon-y; IL-1, interleukin-1; IL-6, interleukin-6; IL-8, interleukin-8; IL-10, interleukin-10; IL-12, interleukin-12; MDSCs, myeloid-derived suppressor cells; MHC, major histocompatibility complex; NK, natural killer; NKT, natural killer T cells; TAM, tumor-associated macrophage; TCR, T cell receptor; TGF-*β*, transforming growth factor-beta; TNF-*α*, tumor necrosis factor-alpha; Tregs, regulatory T cells.

The immune response to tumors involves a variety of immune cells, each contributing to the detection and destruction of cancer cells. Among these, natural killer (NK) cells, cytotoxic T cells, macrophages, and dendritic cells (DCs) play prominent roles (Figure 2).

#### 3.1.1. Cytotoxic T Lymphocytes (CTLs)

CTLs, particularly CD8^+^ T cells, are central to the adaptive immune response against tumors. Upon recognition of TAAs presented by major histocompatibility complex class I (MHC-I) molecules on the tumor cell surface, CTLs are activated and exert their cytotoxic effects by releasing perforin and granzymes, which induce apoptosis in the target tumor cells [33]. Recent research highlights that tumor-infiltrating lymphocytes (TILs), especially CTLs, are linked to improved prognostic outcomes across multiple cancer types, including colorectal cancer and melanoma [34].

#### 3.1.2. NK Cells

NK cells are very important in the innate immune system, providing a rapid response to tumor cells without prior sensitization. These cells are equipped with activating receptors, such as natural killer group 2D (NKG2D), which detect stress-induced ligands on tumor cells, enabling NK cells to target and eliminate them efficiently. Upon activation, NK cells release cytotoxic granules and produce pro-inflammatory cytokines, such as interferon-gamma (IFN-*γ*), to recruit and activate other immune cells [35].

#### 3.1.3. DCs

DCs are specialized antigen-presenting cells (APCs) that are crucial for initiating and regulating adaptive immune responses. By capturing and processing TAAs, DCs present these antigens to T cells, facilitating their activation and differentiation into effector cells [36]. The effectiveness of cancer vaccines and immune checkpoint inhibitors is influenced by the capacity of DCs to present antigens efficiently and initiate T cell activation.

#### 3.1.4. Tumor-Associated Macrophages (TAMs)

TAMs are essential elements of the TME, contributing to both tumor progression and immune responses against cancer. TAMs can have dual roles, either supporting tumor growth and suppression of immune responses or acting as immune effectors that help eliminate tumor cells, depending on their polarization states (M1 or M2) [37]. Targeting TAMs to shift their polarization towards an antitumor phenotype is an area of active research. TAMs will be discussed in detail on its role in TME.

### 3.2. Signaling Pathways Involved in Immune Cell Activation and Tumor Cell Killing

The capacity of the immune system to recognize and eliminate tumor cells is a highly coordinated process involving complex signaling pathways. These pathways regulate the activation, proliferation, and function of immune cells, enabling them to detect and destroy malignant cells [38].

#### 3.2.1. T Cell Receptor (TCR) Signaling Pathway

The TCR signaling pathway is central to the activation of T cells in response to tumors. The TCR complex, present on T cell surface, specifically recognizes antigens that are presented by MHC molecules on APCs [39]. This interaction triggers a cascade of intracellular signaling events. The pathway involves the activation of the CD3 complex, phosphorylation of the Zeta-chain-associated protein kinase 70 (ZAP-70), followed by the recruitment and activation of downstream molecules such as phospholipase C gamma 1 (PLC-*γ*1) and linker for activation of T cells (LATs). This triggers the production of second messengers, such as inositol trisphosphate (IP3) and diacylglycerol (DAG), which elevate intracellular calcium levels and stimulate protein kinase C (PKC), ultimately resulting in the activation of transcription factors, including nuclear factor of activated T cells (NFATs) and NF-*κ*B. A study by Waldman, Fritz, and Lenardo [40] showed that enhancing TCR signaling through genetic engineering could significantly boost the efficacy of adoptive T cell therapy (ACT) in treating solid tumors. Specifically, introducing a constitutively active form of ZAP-70 in T cells led to improved antitumor activity and prolonged survival in melanoma mouse models.

The TCR signaling pathway is modulated by costimulatory and coinhibitory signals, which fine-tune the activation and function of T cells in response to tumors. Costimulatory receptors such as tumor necrosis factor ligand superfamily member 9 (4-1BB; cluster of differentiation 137 [CD137]), cluster of differentiation 40 (CD40), cluster of differentiation 28 (CD28), and tumor necrosis factor receptor superfamily member 4 (OX40; CD134), enhance TCR signaling and promote T cell proliferation and survival. The interaction of CD28 with its ligands, cluster of differentiation 80 (CD80) and cluster of differentiation 86 (CD86), on APCs enhances the TCR signaling cascade, leading to the full activation of T cells. Recent study by Compte et al. [41] demonstrated that an agonistic antibody targeting 4-1BB improved the antitumor activity of ACT in individuals with advanced-stage solid tumors. In contrast, coinhibitory receptors including cytotoxic T lymphocyte-associated protein 4 (CTLA-4), programed cell death protein 1 (PD-1), and lymphocyte activation gene 3 (LAG-3) dampen TCR signaling, leading to reduced T cell activation and the maintenance of self-tolerance. Despite the organized pathways involved in TCR activation, tumors have evolved various mechanisms to disrupt TCR signaling and evade immune detection. These mechanisms include downregulation of MHC molecules, secretion of immunosuppressive cytokines, and induction of regulatory T cells (Tregs) that inhibit T cell activation [42]. Targeting Tregs or neutralizing immunosuppressive cytokines to restore effective TCR signaling is a promising therapeutic approach. A study by Castiglioni et al. [43] found that inhibiting transforming growth factor-beta (TGF-*β*) signaling in conjunction with PD-1 blockade significantly enhanced T cell-mediated tumor rejection in a colorectal cancer murine model.

#### 3.2.2. Phosphatidylinositol 3-Kinase (PI3K)-Akt-Mammalian Target of Rapamycin (mTOR) Pathway

In tumor immunology, PI3K-Akt-mTOR pathway is important in modulating immune cell functions and shaping the TME, influencing the outcomes of antitumor immune responses. PI3K is activated downstream of the TCR and costimulatory receptors such as CD28. Upon activation, PI3K converts phosphatidylinositol 4,5-bisphosphate (PIP2) to phosphatidylinositol 3,4,5-trisphosphate (PIP3), leading to the recruitment of a serine/threonine protein kinase (Akt) to the plasma membrane [44]. Akt is activated through phosphorylation by both 3-phosphoinositide-dependent kinase 1 (PDK1) and the mammalian target of rapamycin complex 2 (mTORC2) complex. Activated Akt inhibits key proteins involved in the apoptotic pathway, thereby, preventing programmed cell death. This kinase mediates survival signals through multiple downstream targets, including the inhibition of factors such as Bcl-2-associated death promoter (BAD), cysteine-aspartic protease (caspase-9), and Forkhead transcription factors, which are directly involved in the apoptotic process. By impeding these proapoptotic elements, Akt ensures the maintenance of cellular integrity and survival under stress conditions, contributing to its pivotal role in tumorigenesis and therapy resistance. It also activates the mTORC1 complex, which controls protein synthesis and cell growth through the phosphorylation of downstream targets like initiation factor 4E binding protein (4E-BP1) and ribosomal S6 kinase (S6K) [45]. PI3K-Akt-mTOR is often dysregulated in cancer, leading to enhanced survival and proliferation of tumor cells. Inhibitors targeting this pathway, such as rapamycin (an mTOR inhibitor), have been explored as potential cancer therapies [46]. A study by Hage and Dormond [47] demonstrated that combining mTOR inhibitors with anti-PD-1 therapy enhanced antitumor immunity and improved survival in a mouse model of glioblastoma. Recent studies have investigated combining PI3K inhibitors with immune checkpoint inhibitors to enhance antitumor immunity. For instance, a clinical trial study by Wu et al. [48] reported that the combination of alpelisib with pembrolizumab (anti-PD-1) improved responses in patients with phosphatidylinositol-4,5-bisphosphate 3-kinase catalytic subunit alpha (PIK3CA)-mutant breast cancer.

#### 3.2.3. NF-*κ*B Signaling Pathway

The nuclear factor kappa B (NF-*κ*B) signaling pathway is an essential regulator of inflammation and immune responses. It is activated by various stimuli, including cytokines, pathogens, and stress signals [49]. NF-*κ*B is normally sequestered in the cytoplasm by inhibitor of *κ*B (I*κ*B) proteins. Upon stimulation, I*κ*B kinases (IKKs) phosphorylate I*κ*Bs, leading to their ubiquitination and subsequent degradation via the proteasome. This releases NF-*κ*B dimers, allowing them to translocate into the nucleus and activate target gene transcription [32]. While NF-*κ*B activity is essential for normal immune function, its dysregulation is implicated in the initiation and progression of cancer. NF-*κ*B activation in cancer cells often promotes tumorigenesis by upregulating the expression of growth factors, antiapoptotic genes, and pro-inflammatory cytokines [34]. For example, NF-*κ*B-induced expression of B-cell leukemia/lymphoma 2 protein (Bcl-2) family proteins inhibits apoptosis, allowing tumor cells to evade cell death and continue proliferating [50]. Conversely, NF-*κ*B also plays a crucial role in antitumor immunity [51]. In immune cells, NF-*κ*B activation enhances the production of pro-inflammatory cytokines and chemokines, which play a key role in recruiting and activating immune effector cells, such as CTLs and NK cells, which are important for tumor killing [52, 53]. Given its dual role, targeting NF-*κ*B to inhibit its pro-tumorigenic functions while preserving or enhancing its role in antitumor immunity is a key therapeutic goal. Recent studies have explored various strategies to modulate NF-*κ*B activity in cancer, with promising results [54].

#### 3.2.4. Janus Kinase (JAK)-Signal Transducer and Activator of Transcription (STAT) Signaling Pathway

The JAK-STAT pathway is critical for signal transmission from cytokine receptors to the nucleus, where it regulates gene expression [55]. Binding of cytokines to their respective receptors triggers associated JAKs activation. These kinases phosphorylate the receptor chains, creating docking sites for STAT proteins. Once activated, the STATs form dimers and move into the nucleus for gene transcription regulation [56]. Different STAT proteins are activated by different cytokines. For example, interleukins-2 (IL-2) triggers the activation of STAT5, a key regulator essential for T cells proliferation and survival, while IFN-*γ* activates STAT1, promoting T helper 1 cells (Th1) differentiation and enhancing the antitumor immune response [57]. The critical role of the JAK/STAT signaling pathway in advancing cancer immunotherapy was also highlighted in a study by Huang and Zappasodi [58], which showed that JAK1/2 inhibition could enhance PD-1 blockade in mouse models of melanoma by reducing tumor-induced immunosuppression. However, some tumors secrete immunosuppressive cytokines like interleukin-10 (IL-10), which activate STAT3 in immune cells, leading to the suppression of antitumor responses and promoting tumor immune evasion [59]. Given its central role in regulating immune responses and tumor progression, the JAK-STAT pathway has emerged as a promising target for cancer immunotherapy. Therapeutic strategies aimed at inhibiting JAK-STAT signaling, including STAT3 decoy oligonucleotides, which compete with endogenous STAT3 for DNA binding, have shown promise in preclinical models of cancer [60].

#### 3.2.5. Fas-FasL Pathway

The Fas–Fas ligand (FasL) pathway is a critical apoptotic signaling pathway used by CTLs and NK cells to induce cell death in target tumor cells. Fas (CD95) is a death receptor present on the surface of various cell types, including tumor cells. FasL is expressed on activated CTLs and NK cells. Binding of FasL to Fas triggers the formation of the death-inducing signaling complex (DISC), which subsequently activates caspase-8 and triggers the apoptotic signaling pathway [61]. Zhu et al. [62] demonstrated that enhancing FasL expression on TILs improved their cytotoxicity against melanoma cells in a mouse model, suggesting a potential therapeutic strategy. However, some tumors develop resistance by downregulating Fas expression or by secreting soluble Fas, which can bind to FasL and prevent it from interacting with membrane-bound Fas on tumor cells [63]. Therapies aimed at restoring Fas expression or enhancing FasL activity are currently being investigated. For example, combining Fas agonists with immune checkpoint blockade has shown to potentiate the immune response against tumors by simultaneously restoring apoptosis and preventing immune suppression [64].

#### 3.2.6. CTLA-4 Pathway

CTLA-4 is an immune checkpoint receptor that plays an important role in downregulating immune responses, particularly by inhibiting T cell activation. Expressed on T cells, CTLA-4 competes with the costimulatory receptor CD28 for binding to B7 molecules (CD80 and CD86) present on APCs, thereby, inhibiting T cell proliferation and function [65]. When CTLA-4 binds to B7, it transmits inhibitory signals that reduce T cell proliferation and cytokine production. The discovery that blocking CTLA-4 can enhance antitumor immunity has led to the development of CTLA-4 inhibitors, such as ipilimumab, the first immune checkpoint inhibitor authorized by the United States Food and Drug Administration (FDA) for melanoma treatment. By blocking CTLA-4 from interacting with B7 ligands, CTLA-4 blockade promotes increased T cell activation and proliferation. Ipilimumab has demonstrated long-term survival benefits in a number of patients, with some achieving complete remission. For instance, a study conducted by Schadendorf et al. [66] found a 10-year survival rate of 21% among advanced melanoma patients who received ipilimumab treatment, demonstrating the potential for durable responses in cancer therapy. Despite its efficacy, CTLA-4 blockade is associated with significant challenges, particularly in terms of irAEs as a result of the broad activation of the immune system, which can lead to autoimmunity and inflammation in various organs [67]. Ongoing research is focused on developing selective CTLA-4 inhibitors that specifically target CTLA-4 on Tregs while sparing effector T cells. This approach could reduce irAEs while maintaining antitumor efficacy. A study by Sobhani et al. [68] reported that a selective use of a CTLA-4 inhibitor demonstrated a significant reduction in tumor growth in a melanoma murine model, while exhibiting a lower incidence of irAEs compared to conventional CTLA-4 blockade therapies.

#### 3.2.7. PD-1/Programed Death-Ligand 1 (PD-L1) Pathway

PD-1 and its ligand, PD-L1, are important components of the immune checkpoint pathway that regulates immune responses. While this pathway is essential for maintaining self-tolerance and preventing autoimmunity, it is often exploited by tumors to evade immune surveillance by expressing PD-L1, which interacts with PD-1 receptors on T cells, leading to the suppression of their function and allowing the tumor to evade immune surveillance [69]. The binding of PD-L1 to PD-1 triggers the recruitment of the phosphatase Src homology-2 protein tyrosine phosphatase (SHP-2), which dephosphorylates critical signaling components within the TCR pathway, ultimately suppressing T cell activation. Tumors can upregulate PD-L1 expression on their surface in response to inflammatory cytokines produced by immune cells. This upregulation allows tumors to effectively “turn off” T cells that recognize and attempt to attack them. By engaging the PD-1 receptor on T cells, PD-L1 expressed by tumor cells inhibits the antitumor immune response [70]. The ability of tumors to exploit the PD-1/PD-L1 pathway is a key strategy to escape immune surveillance, making it a prime target for therapeutic intervention. Therapeutic advancements focused on creating inhibitors that specifically target the PD-1/PD-L1 pathway has significantly advanced cancer immunotherapy, offering new treatment options for patients with various malignancies. Pembrolizumab and nivolumab are two of the most well-known PD-1 inhibitors. These inhibitors disrupt the binding of PD-1 to its ligands, leading to enhanced T cell responses and improved antitumor efficacy [71, 72]. Atezolizumab, durvalumab, and avelumab are PD-L1 inhibitors that disrupt the interaction between PD-1 and PD-L1, as well as PD-L2. These inhibitors have been approved for use in treating cancers including non-small cell lung cancer (NSCLC), bladder cancer, and triple-negative breast cancer (TNBC) [73]. Despite the efficacy of PD-1/PD-L1 inhibitors, some patients do not respond to therapy, and others develop resistance after an initial response. Ongoing research is focused on overcoming these resistance mechanisms to enhance the effectiveness of PD-1/PD-L1-targeted therapies.

### 3.3. TME

The TME is a dynamic and complex ecosystem that plays an important role in cancer development, progression, metastasis, and response to therapies. Comprising diverse cellular and non-cellular components, the TME is highly heterogeneous, and its composition can vary greatly between different tumor types, tumor stages, and even within different regions of the same tumor. This heterogeneity underpins the varied roles that different components of the TME play in supporting or hindering tumor progression. The TME is characterized by altered cell–cell interactions and functional crosstalk between cellular and non-cellular components [74].

#### 3.3.1. Cellular Components

The TME is composed of a diverse array of cellular components that engage in dynamic interactions with each other and tumor cells, collectively shaping cancer progression.

##### 3.3.1.1. Cancer-Associated Fibroblasts (CAFs)

CAFs are specialized fibroblast cells found within the TME and play a crucial role in remodeling the extracellular matrix (ECM) and facilitating tumor progression [75]. CAFs arise from multiple sources, such as mesenchymal stem cells, resident fibroblasts, and epithelial cells undergoing epithelial-to-mesenchymal transition (EMT). They produce a range of cytokines, growth factors, and ECM components that support tumor growth, invasion, and metastasis. For instance, CAFs secrete TGF-*β*, which can promote EMT in cancer cells, enhancing their invasive capabilities [76]. CAFs exhibit diverse functional roles across a wide range of tumor types, including colorectal, prostate, lung, breast, and gastric cancers, each presenting distinct therapeutic challenges. Their ability to dynamically interact with tumor cells and the TME has been increasingly recognized as a critical driver of disease progression and therapeutic outcomes. For example, in estrogen receptor-positive (ER+) breast cancer, emerging evidence highlights the role of CAFs in mediating resistance to endocrine therapies. This resistance is often attributed to the activation of noncanonical signaling pathways, such as the fibroblast growth factor receptor (FGFR) axis, which can sustain tumor cell survival and proliferation despite targeted treatment [76]. In pancreatic ductal adenocarcinoma (PDAC), CAFs constitute a major component of the tumor stroma and have been linked to poor prognosis. They contribute to the dense stromal environment characteristic of PDAC, secrete a wide range of growth factors, cytokines, and chemokines that support tumor proliferation and survival which impedes drug delivery and fosters an immunosuppressive microenvironment [77, 78]. However, emerging evidence suggests that CAFs can also exhibit tumor-suppressive functions, depending on the specific context of the tumor. This duality in CAF behavior presents a significant challenge for cancer immunotherapy, as strategies to target CAFs must be carefully tailored to avoid disrupting their beneficial effects while effectively neutralizing their tumor-promoting activities [79].

##### 3.3.1.2. TAMs

TAMs are a key immune cell population in the TME, often exhibiting a protumorigenic phenotype. TAMs can adopt either an M1 (antitumor) or M2 (protumor) phenotype depending on signals from the TME. M2-polarized TAMs are more prevalent in many tumors and contribute to immunosuppression, angiogenesis, and tissue remodeling, thus, supporting tumor progression [80]. In breast cancer, high densities of M2-polarized TAMs correlate with poor prognosis. These TAMs produce factors such as cytokines (TGF-*β* and IL-10), growth factors and chemokines, that inhibit the activity of cytotoxic T cells and facilitate tumor progression and metastasis [81].

However, TAMs can be reprogramed from a tumor-promoting (M2-like) phenotype to a tumor-suppressing (M1-like) state. This can be achieved by using agents that modulate TAM polarization, such as TLR agonists, CD40 agonists, or IFN-*γ*. Repolarizing TAMs to an M1-like phenotype improves their antigen-presenting capacity and promotes the production of pro-inflammatory cytokines and support antitumor immune responses [82]. A study by Lim et al. [83] illustrated that a CD40 agonist could repolarize TAMs in a pancreatic cancer mouse model, leading to enhanced T cell activation and reduced tumor growth. The study suggests that combining TAM repolarization strategies with immune checkpoint blockade may significantly improve the efficacy of cancer immunotherapy [83]. While targeting TAMs presents a promising approach in cancer immunotherapy, several challenges remain. The heterogeneity and plasticity of TAMs within the TME complicate the development of therapies that can effectively target the protumorigenic functions of TAMs without disrupting their potential antitumorigenic activities [84]. Future research should focus on identifying specific markers and signaling pathways that distinguish protumorigenic TAMs from their antitumorigenic counterparts.

##### 3.3.1.3. Tregs

Tregs, a distinct subset of cluster of differentiation 4-positive (CD4+) T cells, are important in maintaining immune balance by suppressing excessive immune responses. In the TME, they can facilitate immune evasion by suppressing antitumor immune responses through the release of inhibitory cytokines such as IL-10 and TGF-*β*, in addition to direct cell–cell contact. They suppress the activity of effector T cells and NK cells, and other immune cells that are critical for antitumor immunity [85]. Tregs within the ovarian cancer microenvironment suppress the function of TILs, thereby facilitating tumor progression and metastasis [86]. A study by Lee et al. [87] demonstrated that TGF-*β* produced by Tregs in PDAC inhibits the activity of CTLs and promotes tumor growth. Tregs can also suppress antitumor immunity by depriving effector T cells of critical nutrients, including glucose and amino acids, within the TME. This metabolic competition can lead to the deprivation of these nutrients for effector T cells, thereby, inhibiting their proliferation and function. Given the role of Tregs in promoting immune evasion in the TME, targeting these cells has become a promising approach in cancer immunotherapy. One approach to targeting Tregs is to selectively deplete them from the TME. Anti-CD25 (cluster of differentiation 25) antibodies, which target the alpha chain of the IL-2 receptor (CD25) expressed on Tregs, have been investigated as a means to deplete Tregs. However, this approach has limitations due to the expression of CD25 on activated effector T cells, leading to potential off-target effects [88].

##### 3.3.1.4. Myeloid-Derived Suppressor Cells (MDSCs)

MDSCs are heterogeneous group of immature myeloid cells that suppress NK cell and TCR function in cancer patients, as well as other pathological states associated with stress or chronic inflammation. They promote angiogenesis and tumor growth, drive metastasis progression and the resistance towards immunotherapy [89]. In the TME, MDSCs release arginase, nitric oxide and reactive oxygen species (ROS) to inhibit T cell activation/proliferation by depleting essential nutrients like arginine and cysteine from the environment as factors that impair T cell metabolism/function [90]. Additionally, MDSCs are capable of upregulating immune checkpoint molecules like PD-L1, which impairs T cell function by interacting with PD-1 receptors on T cells [91]. They also promote Tregs development and enhance the immunosuppressive environment within the TME [92]. MDSCs could inhibit NK cell function by releasing TGF-*β*, leading to a reduced expression of NK cell activating receptors [93]. This inhibition of NK cell activity facilitates immune evasion by tumor cells, emphasizing the need to target MDSCs to restore NK cell activity and improve antitumor immunity. Emerging evidence suggests that MDSCs can be targeted with drugs to inhibit their protumorigenic and immunosuppressive functions, thereby increasing the efficacy of checkpoint inhibitors [94].

##### 3.3.1.5. Endothelial Cells

Endothelial cells are the major components of blood vessels within the TME, where they significantly contribute to the process of tumor angiogenesis. This process essential for providing tumors with nutrients and oxygen [95]. Tumor endothelial cells are often abnormal, leading to disorganized and leaky vasculature. They respond to proangiogenic signals, including vascular endothelial growth factor (VEGF) secreted by tumor cells and stromal cells, leading to the development of new blood vessels that facilitate tumor expansion and metastasis [95]. In glioblastoma, one of the most vascularized tumors, high levels of VEGF contribute to the development of an extensive vascular network that support rapid tumor growth. Antiangiogenic therapies targeting VEGF signaling, such as bevacizumab, have been developed to disrupt this process [96]. Endothelial cells also have the capacity to express adhesion molecules that preferentially facilitate the entry of immunosuppressive cells such as Tregs and MDSCs, while limiting the infiltration of T cells into the TME [97]. This preferential migration of immune cells contributes to creating an immunosuppressive environment, which promotes tumor progression and undermines the effectiveness of immunotherapies.

##### 3.3.1.6. Cancer-Associated Adipocytes (CAAs)

CAAs are adipocytes that are found in the TME, particularly in proximity to solid tumors including those in the breast, ovary, and pancreas. They interact closely with tumor cells, playing a pivotal role in driving tumor advancement. CAAs contribute to the metabolic reprograming of cancer cells, providing them with the necessary energy for growth and survival [98]. Recent studies have shown that CAAs can promote immune escape within the TME by secreting various adipokines and inflammatory cytokines. These secretions can suppress cytotoxic T cell function and enhance the recruitment of immunosuppressive cells such as MDSCs and Tregs. Evidence suggests that CAAs play important roles in developing resistance to immunotherapy through the secretion of specific factors that protect tumor cells from immune attack. Dosch et al. [99] demonstrated that in pancreatic cancer, CAAs produce interleukin-6 (IL-6), which triggers the activation of the STAT3 signaling pathway in tumor cells. This activation leads to an upregulation of survival pathways and a reduction in the efficacy of immune checkpoint inhibitors [99]. The study suggested that targeting IL-6 signaling in CAAs could enhance the response to immunotherapy.

#### 3.3.2. Noncellular Components

The noncellular components, also known as the ECM and various soluble factors, contribute significantly to the structural integrity of the TME and influence cell behavior.

##### 3.3.2.1. ECM

The ECM consists of an intricate network of proteins and macromolecules that provide both structural integrity and biochemical signals to surrounding cells. In the TME, key constituents of the ECM include fibronectin, collagen, laminin, elastin, proteoglycans, and glycosaminoglycans. In tumors, collagen is often overproduced and aberrantly organized, leading to increased tissue stiffness, which can facilitate tumor growth and metastasis [100]. Tumor cells often exploit fibronectin and laminin to facilitate invasion by interacting with integrins and other cell surface receptors [101]. Proteoglycans, such as heparan sulfate and hyaluronan, are critical for maintaining ECM structure and regulating cell signaling. In tumors, these molecules can modulate growth factor availability and receptor activity, influencing tumor growth and angiogenesis [102].

ECM remodeling is a dynamic process that occurs in the TEM involving the degradation, synthesis, and reorganization of ECM components. This process is driven by various enzymes, such as matrix metalloproteinases (MMPs), which are often upregulated in tumors. MMPs are group of proteolytic enzymes responsible for breaking down ECM components, which plays a key role in promoting tumor invasion and metastasis. For example, MMP-2 and MMP-2 and MMP-9 target type IV collagen, a crucial component of the basement membrane, enabling its degradation. This allows tumor cells to invade surrounding tissues [103]. The ECM functions not only as a structural framework but also as an active regulator of cellular behavior through various signaling pathways. These interactions are primarily mediated by surface receptors, including integrins, syndecans, and discoidin domain receptors. In pancreatic cancer, the interaction between integrins and the ECM component laminin promotes the activation of the mitogen-activated protein kinase/extracellular signal-regulated kinase (MAPK/ERK) signaling cascade, which enhances tumor cell proliferation and resistance to apoptosis [104].

##### 3.3.2.2. Exosomes and Extracellular Vesicles (EVs)

Exosomes and EVs are membrane-bound particles that play crucial roles in cell-to-cell communication within the TME. These vesicles, released by both tumor and stromal cells, transport a range of biomolecules, such as nucleic acids, lipids, and proteins, which modulate the activity and function of recipient cells [105]. Tumor-derived exosomes (TDEs) can alter the ECM, promoting tumor cell invasion and progression. For instance, exosomes derived from highly metastatic melanoma cells have been demonstrated to carry MMPs and integrins, which degrade the ECM and promote metastatic spread [106]. In breast cancer, exosomes carrying microRNA-222 have been associated with the emergency of resistance to tamoxifen, a commonly used hormone therapy. These exosomes can transfer drug resistance to sensitive cells, highlighting the role of EVs in promoting therapy-resistant tumor phenotypes [107]. As research in tumor immunotherapy advances, gaining a comprehensive understanding of how exosomes and EVs modulate the TME will be crucial for the development of innovative cancer therapies.

##### 3.3.2.3. Hypoxia and Metabolism

Hypoxia, a condition of reduced oxygen levels, is a common feature of the TME and drives metabolic adaptations in cancer cells. Tumor cell rapid proliferation and insufficient blood supply create a hypoxic environment, which in turn triggers a range of adaptive responses [108]. These responses, coupled with metabolic reprograming, enable tumor cells to survive and thrive under hostile conditions. In hypoxic environments, hypoxia-inducible factor 1 alpha (HIF-1*α*) becomes stabilized and moves into the nucleus, where it forms a dimer with hypoxia-inducible factor 1 beta (HIF-1*β*). This dimerization triggers the activation of genes involved in angiogenesis, as well as the upregulation of VEGF, and facilitates metabolic reprogramming [109]. This adaptation allows tumor cells to survive in low-oxygen environments by stimulating the development of new blood vessels and switching to anaerobic metabolic pathways. In glioblastoma, a highly aggressive brain tumor, stabilization of HIF-1*α* under hypoxic conditions has been associated with elevated VEGF expression, which promotes angiogenesis and tumor growth. This process contributes to the characteristic necrotic and hypoxic regions within glioblastomas [110]. Given the central role of hypoxia and metabolism in driving tumor progression, targeting these processes represents a promising therapeutic strategy, including the use of HIF inhibitors, hypoxia-activated prodrugs (HAPs), and agents that normalize tumor vasculature to improve oxygen delivery.

## 4. Limitations and Challenges in Current Tumor Immunology and Immunotherapy

Tumors develop multiple strategies to circumvent immune detection, enabling immune evasion, and facilitating cancer progression. Some of the challenges with current immunotherapy options include:

### 4.1. TME

One of the major limitations in current tumor immunology is the complexity of the TME. This environment often promotes immunosuppression, allowing tumors to evade immune detection and destruction. For instance, Tregs and MDSCs within the TME can inhibit CTLs activity, reducing the effectiveness of immunotherapies including immune checkpoint inhibitors [111]. Tumor cells can also reduce the expression of TAAs, making them invisible to the immune system. For example, melanoma cells may lose expression of melanoma-associated antigen recognized by T cells (MART-1) and glycoprotein 100 (gp100), reducing their susceptibility to immune attack [112].

### 4.2. Immunotherapy Resistance

Another challenge is the development of resistance to immunotherapy. Tumors can become resistant through various mechanisms, including the loss of antigen presentation, upregulation of alternative immune checkpoints, and alterations in signaling pathways [113]. These resistance mechanisms may reduce the long-term effectiveness of therapeutic interventions like PD-1/PD-L1 inhibitors, necessitating the development of combination therapies or new therapeutic targets.

### 4.3. Heterogeneity and Biomarker Identification

Tumor heterogeneity, both intertumoral and intratumoral, poses a significant challenge in immunotherapy. The genetic and epigenetic diversity within a single tumor can result in variable expression of antigens, leading to differential responses to immunotherapy [114]. This heterogeneity complicates the identification of reliable biomarkers for predicting response to treatment. Although PD-L1 expression is used as a predictive biomarker in anti-PD-1/PD-L1 therapies, its predictive value is not absolute, and responses can occur in PD-L1-negative tumors [115]. The identification of robust biomarkers is essential for patient stratification and optimizing treatment outcomes. However, the dynamic nature of the immune response and tumor evolution during therapy further complicates biomarker development [116].

### 4.4. Adverse Effects of Immunotherapy

Despite the revolutionary advancements immunotherapy has brought to cancer treatment, leading to significantly better outcomes for numerous patients, it is associated with a variety of irAEs. These adverse effects can impact multiple organ systems and vary in severity, presenting challenges in clinical management [117]. Dermatologic toxicities are among the most common irAEs reported in patients undergoing immunotherapy. These typically include rash, pruritus (itching), and vitiligo. In a study involving patients receiving anti-PD-1 treatments, such as nivolumab, approximately 20%–40% experienced pruritus and rash as side effects [9]. Another adverse effect is endocrine irAEs, which are common and often require long-term management due to their potential to cause irreversible damage to endocrine glands. Pulmonary irAEs, particularly pneumonitis, are among the most serious complications associated with immunotherapy. Lung tissue inflammation, known as pneumonitis, occurs in approximately 5%–10% of undergoing treatment with PD-1/PD-L1 inhibitors [118]. Neurological irAEs are relatively rare but can be severe, affecting the peripheral and central nervous systems. Peripheral neuropathy, characterized by numbness, tingling, and weakness in the limbs, has been reported in patients receiving immunotherapy. Larkin et al. [119] reported that around 1%–2% of patients treated with nivolumab developed this condition. Cardiovascular irAEs, including myocarditis and pericarditis, are uncommon but can be life-threatening if not promptly identified and treated. Myocarditis, an inflammation of the heart muscle, has been observed in less than 1% of individuals treated with checkpoint inhibitors, but it carries a high mortality rate [120].

## 5. Prophylactic and Therapeutic Applications of Immunological Products on Tumor Cells

**Figure 3:**
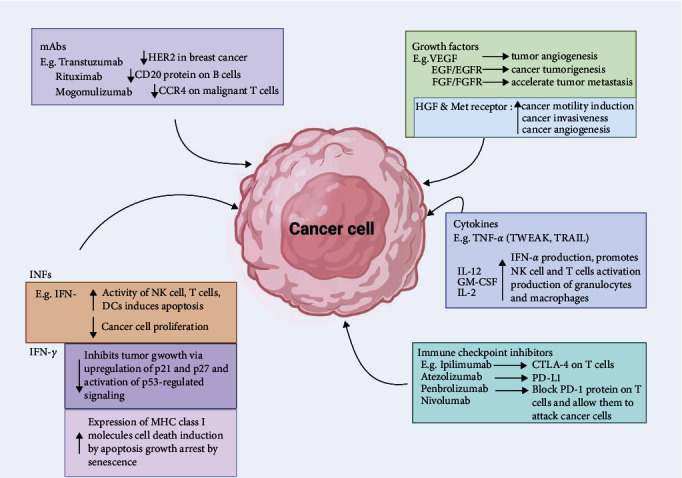
Immunological products involved in cancer therapy. CCR4, C–C chemokine receptor type 4; CTLA-4, cytotoxic T lymphocyte-associated protein 4; DC, dendritic cell; EGF, epidermal growth factor; EGFR, epidermal growth factor receptor; FGF, fibroblast growth factor; FGFR, fibroblast growth factor receptor; GM-CSF, granulocyte-macrophage colony-stimulating factor; HER2, human epidermal growth factor receptor 2; HGF, hepatocyte growth factor; IFN-*α*, interferon-alpha; IFN-*γ*, interferon-gamma; INFs, interferons; IL-2, interleukin-2; IL-12, interleukin-12; mAbs, monoclonal antibodies; MHC, major histocompatibility complex; NK, natural killer; PD-1, programed cell death protein 1; PD-L1, programed death-ligand 1; TRAIL, TNF-related apoptosis-inducing ligand; TWEAK, TNF-like weak inducer of apoptosis; VEGF, vascular endothelial growth factor.

Immunological products (Table 2) consist of a group of biopharmaceuticals capable of modulating the recipient's immune status such that the recipient is protected from infectious or noninfectious diseases. Some examples of such products currently available against cancer cells are monoclonal antibodies, cancer vaccines, immune checkpoint inhibitors, antibody-targeted therapeutics, diagnostics, and immune system modulators (Figure 3) [59, 131].

### 5.1. Cancer Vaccines

Cancer vaccines are biological preparations that stimulate the immune system to generate T and B lymphocytes to target tumor cells. They can be either prophylactic or therapeutic, made of peptides or recombinant DNA.

#### 5.1.1. Cancer Prevention Vaccines

Prophylactic cancer vaccines are designed to prevent cancer by immunizing individuals against infectious agents that are known to cause cancer. The most successful examples of prophylactic cancer vaccines are those targeting viruses including Epstein-Barr Virus, human papillomavirus (HPV), and hepatitis B virus (HBV), which are associated with liver cancer, cervical cancer, and Burkitt's lymphoma, respectively [130, 132]. Lei et al. [133] observed a significant decline in the prevalence of HPV infections and precancerous lesions in populations with high vaccination coverage, indicating that broad implementation of HPV vaccination could significantly decrease the overall burden of cervical cancer worldwide. A long-term study conducted in Taiwan, where the HBV vaccine was implemented as part of the national immunization program, revealed that incidence of hepatocellular carcinoma was significantly lower in vaccinated individuals compared to those who were not vaccinated [134]. FDA-approved vaccines like Cervarix, Gardasil, Gardasil-9, and hepatitis B vaccine (recombinant), adjuvanted (HEPLISAV-B) help prevent the development of various cancers caused by HPV and HBV [122, 123].

#### 5.1.2. Therapeutic Cancer Vaccines (TCVs)

TCVs activate the immune system by targeting specific TAAs, aiming to decrease tumor load and prevent recurrence. TCVs can induce the regression of tumors, eradicate residual disease, and create long-term antitumor memory while preventing nonspecific or adverse reactions [135]. TCVs can be developed using tumor cells or their components, as well as from the patient's DCs [126]. An FDA-approved TCV, Sipuleucel-T (Provenge), made from personalized stimulated DCs is used to manage people with metastatic prostate cancer [136]. Clinical studies have shown that Sipuleucel-T significantly enhances overall survival in individuals diagnosed with metastatic castration-resistant prostate cancer (mCRPC), although its impact on tumor size and progression-free survival is modest. Kantoff et al. [137] reported that patients receiving Sipuleucel-T therapy had an increase in median overall survival of around 4.1 months when compared to the placebo group, highlighting the potential of therapeutic vaccines in extending the lives of cancer patients. Recent advances in genomics have enabled the development of personalized cancer vaccines that focus on neoantigens, which are proteins that arise from tumor-specific mutations. These vaccines are tailored to the unique mutational landscape of an individual's tumor, making them highly specific to the tumor cells while sparing normal tissues. Yarchoan et al. [138] highlighted that a personalized neoantigen vaccine with pembrolizumab induced robust T cell responses and led to sustained clinical improvements in patients with advanced hepatocellular carcinoma, representing a significant step forward in personalized cancer immunotherapy. Other TCVs being explored include the herpes simplex virus type 1 vaccine talimogene laherparepvec (T-VEC) and the Bacillus Calmette-Guérin (BCG) vaccine [129, 139].

### 5.2. Monoclonal Antibodies (mAbs)

mAbs are engineered proteins that specifically bind to antigens present on tumor cell surfaces. They can mediate tumor cell death through several pathways, such as complement activation, antibody-dependent cellular cytotoxicity (ADCC), direct inhibition of growth signals, blocking of growth factor receptors to halt tumor cell multiplication, and formation of anti-idiotype antibodies to enhance the patient's immune response [140]. mAbs can also be engineered to bind to two epitopes, known as bispecific monoclonal immunoglobulins [141].

Immune checkpoint inhibitors, a type of mAb, work by blocking immune system checkpoint proteins, allowing T lymphocytes to recognize and destroy cancerous cells [128, 142]. FDA-approved immune checkpoint inhibitors target specific proteins such as PD-1, PD-L1, and CTLA-4 and have shown efficacy in treating various cancers, including cervical, breast, colon, bladder, head, neck, lung, liver cancers, and Hodgkin lymphoma [143]. For instance, in NSCLC, the expression of PD-L1, a TAA, on cancer cells is linked to mechanisms of immune escape. The application of PD-1 inhibitors, including pembrolizumab, have been shown to significantly improve survival rates in patients with PD-L1-positive NSCLC [144]. Immune checkpoint inhibitors, including nivolumab and pembrolizumab, which target PD-1, along with therapies aimed at PD-L1, including atezolizumab, have demonstrated significant therapeutic efficacy in the management of melanoma, NSCLC, and renal cell carcinoma [116]. Gandhi et al. [145] revealed that pembrolizumab combined with chemotherapy led to a substantial improvement in survival outcomes for patients with metastatic non-small cell lung cancer NSCLC, establishing this combination as a standard first-line treatment. Ipilimumab, an antibody targeting CTLA-4, was the first immune checkpoint inhibitor authorized for cancer therapy and has been particularly effective in melanoma. A study by Hodi et al. [146] demonstrated that treatment with ipilimumab resulted in increased overall survival in patients diagnosed with metastatic melanoma.

As of October 2023, the FDA, European medicines agency (EMA), and other agencies have approved 91 monoclonal antibodies for cancer treatment, with many more in development. Some examples of FDA-approved monoclonal antibodies for cancer treatment as shown in Table 3 include denosumab, ipilimumab, trastuzumab, blinatumomab, ibritumomab, ofatumumab, pembrolizumab, rituximab, and obinutuzumab [147]. These mAbs and immune checkpoint inhibitors have transformed cancer therapy by boosting the immune system's capacity to recognize and destroy malignant cells.

### 5.3. Adoptive T Cell Therapy (ACT)

ACT is a form of cancer immunotherapy that involves engineering the patient's T lymphocytes to fight cancer [149]. This approach entails the *ex vivo* proliferation of T cells that selectively target tumor antigens, followed by their infusion into the patient. The most direct form of ACT involves the expansion of TILs derived from a patient's tumor.

#### 5.3.1. TILs Therapy

TILs are T cells that naturally migrate into and accumulate within tumor tissues, suggesting they have some degree of tumor specificity. They include different subsets of B cells, T cells, and NK cells, which can either promote or inhibit tumor progression. Once isolated, these cells can be expanded in large numbers in the laboratory using cytokines such as interleukin-2 before being reinfused into the patient [150]. TIL therapy has demonstrated promising outcomes in melanoma patients and patients with cervical squamous cell carcinoma and cholangiocarcinoma [151, 152]. The National Cancer Institute [153] is conducting a phase II clinical trial to explore the use of TIL therapy in metastatic cancers. To enhance the specificity and efficacy of T cells in targeting tumors, genetic modifications can be introduced to enable the expression of receptors specific to tumor antigens [152]. Adoptive T cell therapies (ACT) utilize two primary types of these engineered T cells: chimeric antigen receptor (CAR) T cells and TCR T cells.

#### 5.3.2. CAR T Cells

CAR T cells are synthetic and combine antigen recognition and T cell activation into a single receptor, enabling them to specifically recognize and target tumor antigens directly, bypassing the need for traditional antigen processing by tumor cells [154]. CAR T cell therapy has transformed the treatment of B cell malignancies, including acute lymphoblastic leukemia (ALL) and certain types of lymphoma. CAR T cells targeting CD19, a protein commonly expressed on B cells, have demonstrated remarkable clinical responses in patients who have relapsed or failed to respond to conventional treatments. For instance, in a landmark clinical study reported a 90% complete remission rate in pediatric and young adult individuals with relapsed or refractory ALL following CD19-specific CAR T cell therapy. This groundbreaking result contributed to the FDA's initial approval of tisagenlecleucel, marking the first CAR T cell therapy authorized for use in these patients [155]. It is being researched in various other cancer treatment settings and may become more widely used due to ongoing clinical research [127].

#### 5.3.3. TCR T Cells

TCR T cells are altered to express TCRs that specifically recognize antigens presented by MHC molecules on tumor cell surface. Unlike CARs, TCRs are capable of detecting intracellular antigens that are displayed on the tumor cell surfaces via MHC molecules, broadening the range of targetable antigens [156].

TCR T cells directed against antigens like New York esophageal squamous cell carcinoma 1 (NY-ESO-1), a cancer-testis antigen, have shown clinical activity in patients with melanoma and synovial sarcoma. In a clinical trial study involving patients with advanced synovial sarcoma, over 50% of patients responded positively to TCR T cells. This trial highlighted the potential of TCR T cells to recognize and target intracellular antigens within solid tumors [157].

Despite the significant progress, several challenges and limitations must be addressed to fully achieve the potential of ACT, including toxicities and tumor antigen escape. Overcoming these challenges requires innovative approaches to enhance T cell trafficking, persistence, and function within the TME [158] Determining the optimal dose of transferred T cells is also important maximize therapeutic outcomes, and continuous monitoring of the patient's response to treatment is essential to ensure efficacy [159].

### 5.4. Cytokines

Cytokines are small proteins produced by leucocytes, that play crucial roles in regulating the body's innate immune responses as well as enhancing the immune system's capacity to overcome cancer [160]. Their dual role in enhancing immune responses and directly targeting tumor cells has led to substantial advancements in both prophylactic and therapeutic applications. Cytokines applied in cancer immunotherapy include:

#### 5.4.1. Interferons (IFNs)

IFNs, particularly IFN-*α* and IFN-*β*, are critical in the body's defense against viral infections. They enhance the antiviral state of cells, promote the activation of NK cells, and enhance antigen presentation to T cells. IFN-*α* has been used prophylactically in individuals at high risk of viral infections, including those with chronic hepatitis B and C, to prevent the progression of liver disease and reduce the risk of hepatocellular carcinoma [161]. For example, IFN-*α* has been employed in patients with melanoma and renal cell carcinoma after primary treatment to prevent relapse. Ives et al. [162] recently reported that adjuvant IFN-*α* therapy led to a significant improvement in relapse-free survival among patients with high-risk melanoma, suggesting its role in preventing disease recurrence. Recent advancements in the therapeutic use of IFN-*α* and IFN-*β* include the use of pegylated IFNs, which offer extended half-life and improved patient convenience. Jia et al. [163] showed that pegylated IFN-*α* could effectively treat advanced melanoma and induce durable responses with manageable side effects.

#### 5.4.2. IL-2

IL-2, commonly referred to as T cell growth factor, aids in the proliferation and mobilization of body leucocytes (including natural killer T cells (NKT) and NK cells) against cancer and also aids the B cells in generating substances capable of killing cancer cells [164, 165]. Recent studies have explored its prophylactic use in maintaining remission in patients with hematologic malignancies. Maharaj et al. [166] demonstrated in their study that administering low-dose IL-2 could prolong remission in patients with chronic lymphocytic leukemia (CLL) by enhancing the activity of residual T cells and NK cells. High-dose IL-2 therapy has been fundamental in the treatment of metastatic melanoma and renal cell carcinoma, primarily due to its capacity to enhance the expansion and activation of cytotoxic T cells and NK cells. Recent research has focused on improving IL-2 therapy by combining it with other immunotherapies. Kleef et al. [167] highlighted the effectiveness of combining IL-2 with immune checkpoint inhibitors, resulting in enhanced response rates and prolonged survival outcomes in patients with metastatic melanoma.

#### 5.4.3. Interleukin-12 (IL-12)

IL-12 is a highly effective cytokine that enhances the immune response against tumors by promoting Th1 differentiation and activating CTLs. Recent clinical studies have focused on the potential of IL-12 in gene therapy, both as a standalone treatment and when used in conjunction with checkpoint inhibitors. Liao et al. [168] established in their study that intratumoral administration of an IL-12-encoding plasmid, combined with PD-1 inhibitors, led to significant tumor regression and improved immune cell infiltration.

#### 5.4.4. Tumor Necrosis Factor-*α* (TNF-*α*)

TNF-*α* triggers apoptosis predominantly in malignant cells, while leaving normal cells unaffected. Recent studies have explored TNF-*α* -based therapies in combination with other agents to overcome resistance. Preclinical studies have suggested that TNF-*α* can augment antitumor immunity by increasing the expression of MHC molecules and costimulatory factors on APCs. Research by Zhang et al. [169] revealed that administering TNF-*α* in a murine cancer model significantly boosted DC activity, resulting in improved T cell responses, indicating its potential as a prophylactic agent to boost immune surveillance. Historically, TNF-*α* was used in high-dose regimens to treat localized cancers, such as sarcomas. Despite its efficacy, high-dose TNF-*α* therapy was limited by severe side effects. Recent studies have focused on optimizing its delivery and combining it with other treatments. Deroose et al. [170] showed that low-dose TNF-*α* combined with radiation therapy led to significant tumor regression in patients with soft tissue sarcoma, showing that controlled administration of TNF-*α* can enhance therapeutic outcomes while minimizing adverse effects. Combining TNF-*α* with immune checkpoint inhibitors has also emerged as a strategy to augment antitumor immune response. In patients with metastatic melanoma, this combination has been shown to enhance the effectiveness of PD-1 inhibitors through synergistic mechanisms, leading to higher response rates and prolonged survival [171]. Despite the therapeutic potential of TNF-*α* in cancer treatment, significant obstacles remain, including toxicity, development of resistance, and inconsistent patient responses, which require further investigation and optimization. Combining TNF-*α* with other therapeutic approaches, including targeted therapies and immune checkpoint inhibitors, as well as identifying biomarkers that predict response, and tailoring treatments accordingly will be crucial for improving outcomes [172].

### 5.5. Hematopoietic Growth Factors

Hematopoietic growth factors are used as an adjunct therapy in cancer management to reduce the adverse effects of cancer treatment. They can rejuvenate anemia, neutropenia, and thrombocytopenia caused by cancer chemotherapy [173]. Examples of hematopoietic growth factors include erythropoietin (red blood cells enhancer), interleukin-11 (IL-11; platelets enhancer), erythrocyte stimulating agents (ESAs), thrombopoietin mimetics, granulocyte-macrophage colony-stimulating factor (GM-CSF), and granulocyte colony-stimulating factor (G-CSF) which improve leucocyte counts, leading to a reduced chance of infections. GM-CSF and G-CSF mobilize the T lymphocytes against cancer cells. G-CSF and GM-CSF are also essential regulators of angiogenesis especially in pathological conditions, and they serve as a link between angiogenesis and hematopoiesis [174]. Also, probiotics have been found useful in cancer immunomodulation [175]. Recent research indicate that G-CSF administration markedly lower the incidence of febrile neutropenia and hospitalizations. A study by Kuderer et al. [176] demonstrated that G-CSF prophylaxis effectively reduced infection rates and improved the quality of life in patients undergoing chemotherapy for breast cancer. Therapeutic administration of IL-11 has demonstrated effectiveness in enhancing platelet regeneration and mitigating bleeding risks in patients receiving chemotherapy [177].

## 6. Conclusion

Over the past few decades, extensive preclinical and clinical research has greatly advanced our understanding of the complex interplay between the immune system and cancer. The application of various products of immunotherapy has revolutionized traditional cancer, leading to notable enhancements in patient survival and quality of life, especially when compared to traditional approaches like surgery, chemotherapy, and radiotherapy.

## 7. Future Prospective

Cancer immunotherapy faces significant challenges, particularly due to resistance that arises from the complex interplay between the immune system, tumor cells, and the microenvironment. Additionally, identifying the ideal dosage for different stages of cancer, identifying, and designing the ideal personalized immunological products that will confer genuine benefits on the cancer patient and not just putting pressure on the host immune balance without attacking the cancer cells. To address these challenges, novel products should be designed to enhance immunogenicity by directly attacking tumor cells and altering the microenvironment. Research efforts should focus on better understanding the TME, overcoming resistance mechanisms, and developing novel biomarkers for more precise patient selection. Additionally, improving the management of irAEs is essential to ensuring the safety and maximizing the efficacy of immunotherapies. clustered regularly interspaced palindromic repeats (CRISPRs)/CRISPR-associated protein 9 (Cas9) gene editing technology has demonstrated potential in targeting hereditary cancers, with ongoing clinical studies expected to further confirm its therapeutic efficacy. In personalized cancer therapy, further research into the genomics, and proteomics of neoantigens and the immunopharmacological potentials of neoantigen-based vaccines will offer greater precision in combating malignancies.

## Figures and Tables

**Table 1:** Types of cancer therapy.

Type of therapy	Mechanism	Pros	Cons	References
Surgery	Physical removal of the tumor and potentially affected surrounding tissues and lymph nodes.	• Often curative for localized cancers. • Immediate tumor removal. • Minimal systemic side effects.	• Invasive, risk of infection, and complications. • Not suitable for advanced/metastatic cancers. • Potential for recurrence if complete resection is not achieved.	[10, 11]

Radiotherapy	Utilizes high-energy ionizing radiation, such as X-rays or gamma rays, to induce DNA damage in cancer cells, ultimately leading to their destruction.	• Effective for localized tumors. • Noninvasive. • Modern precision techniques reduce collateral damage.	• Can damage healthy tissue, causing long-term side effects like fibrosis. • Risk of secondary malignancies due to radiation exposure. • Not all cancers respond well to radiation.	[12, 13]

Chemotherapy	Utilizes cytotoxic agents that specifically target and interfere with the proliferation of rapidly dividing cancer cells, disrupting their growth and division.	• Effective for treating metastatic and systemic cancer. • Can be used in combination with surgery or radiation. • Multiple drug classes for tailored therapy.	• High toxicity. • It nonselectively targets rapidly proliferating cells, including healthy tissues such as bone marrow and hair follicles. • Risk of drug resistance. • Common adverse effects include severe nausea, alopecia, and immunosuppression, and increased infection risk.	[14, 15]

Targeted therapy	Focuses on cancer-specific pathways critical for cancer cell growth and survival (e.g., tyrosine kinases, HER2, EGFR).	• Precision treatment with a reduced incidence of adverse effects relative to conventional chemotherapy. • Highly effective for cancers with specific genetic mutations (e.g., CML, NSCLC). • Can be combined with other therapies.	• Tumors may develop resistance through alternative pathways. • Limited to cancers with specific molecular targets. • Expensive and not accessible for all patients.	[16, 17]

Hormone therapy	Involves blocking the hormones (e.g., estrogen and testosterone) that fuel certain cancers (e.g., breast and prostate cancer).	• Highly effective for hormone receptor-positive cancers. • Fewer incidence of adverse effects relative to chemotherapy. • Can be used long-term to control cancer growth.	• Only effective in hormone-dependent cancers. • Tumors may acquire resistance over time. • Adverse effects such as hot flashes, osteoporosis, and cardiovascular issues.	[18, 19]

Stem cell transplantation	Involves the transplantation of healthy stem cells to replace bone marrow that has been damaged or destroyed.	• Allows for high-dose chemotherapy that might otherwise be too toxic. • Effective in blood cancers like leukemia and lymphoma. • Can be curative for certain blood cancers.	• High-risk procedure with potential complications such as GVHD. • Requires precise matching of donor cells. • Long recovery time and high cost.	[20, 21]

PDT	Uses photosensitizing agents and light to induce the production of ROS, leading to targeted cancer cell destruction.	• Minimally invasive and highly targeted. • Less damage to surrounding tissues. • Can be repeated multiple times if necessary.	• Effective mainly for surface or accessible tumors (e.g., skin and esophagus). • Limited depth of light penetration. • Potential for photosensitivity reactions.	[22]

Gene therapy	Introduces genetic material into cancer cells to either kill them or boost the immune response against the tumor.	• Potential to target cancer at the genetic level. • Offers hope for previously untreatable cancers. • May provide long-term remission by correcting mutations.	• Experimental with ongoing clinical trials. • Risk of off-target effects and mutations. • High cost and technical complexity.	[23]

Immunotherapy	Uses the body's immune system to precisely target and eliminate cancer cells.	• Can lead to long-term remission in metastatic cancers like melanoma. • Fewer adverse effects compared to traditional chemotherapy. • Effective in cancers previously deemed untreatable. • Long-lasting response. • Immunological memory and reduced risk of recurrence. • Can identify and target a wide range of tumor antigens. • Combinatorial potential with other therapies.	• Not all patients respond to immunotherapy. • Immune-related side effects (e.g., colitis, pneumonitis, and endocrine disorders).	[24, 25]

Abbreviations: CML, chronic myeloid leukemia; EGFR, epidermal growth factor receptor; GVHD, graft-versus-host disease; HER2, human epidermal growth factor receptor 2; NSCLC, non-small cell lung cancer; PDT, photodynamic therapy; ROS, reactive oxygen species.

**Table 2:** Antitumor immunological products [121–130].

Immunological product	Function	Type of cancer treated/prevented
HEPLISAV-B	Preventive	HBV-related liver cancer

Cervarix	Preventive	HPV-related anal, cervical, vulvar, head and neck, penile, and vaginal cancers

Gardasil-4	Preventive	HPV-related anal, cervical, vulvar, head and neck, penile, and vaginal cancers

Gardasil-9	Preventive	HPV-related anal, cervical, vulvar, head and neck, penile, and vaginal cancers

Hepatititis B vaccine (HEPLISAV-B)	Preventive	HBV-related hepatocellular carcinoma

Bacillus Calmette–Guérin	Treatment	Bladder cancer

T-VEC, or Imlygic	Treatment	Melanoma

Sipuleucel-T (Provenge)	Treatment	Prostate cancer

Monclonal antibodies	Identify and bind to specific antigenic proteins	Denosumab for prostate cancer
		Ipilimumab for advanced melanoma
		Trastuzumab for breast cancer
		Blinatumomab for leukamia

Immune checkpoint inhibitors	Inhibition of immune checkpoint proteins	PD-1 inhibitors (pembrolizumab, nivolumab, and camiplimab)
		PD-L1 inhibitors (durvalumab, atrzolimumab, and avelumab)
		CTLA-4 inhibitor (ipilimumab)

Tumor infiltrating lymphocytes therapy	Attack cancer cells	Melanoma, cervical squamous cell carcinoma and cholangiocarcinoma

Chimeric antigen receptor T cell therapy	Attacks cancer cells	Leukemia that has not responded to initial chemotherapy

Type I INFs	Regulate transcription of cytokine genes as well as activate immune effector cells	IFN-*α* for melanoma, leukemia and non-Hodgin lymphoma

Hematopoietic growth factors	Reduce the adverse effects of cancer treatment	ErythroA, G-CSF, GM-CSF

Abbreviations: CTLA-4, cytotoxic T lymphocyte-associated protein 4; G-CSF, granulocyte colony-stimulating factor; GM-CSF, granulocyte-macrophage colony-stimulating factor; HBV, hepatitis B virus; HEPLISAV-B, hepatitis B vaccine (recombinant), adjuvanted; HPV, human papillomavirus; IFN-*α*, interferon-alpha; INFs, interferons; PD-1, programed cell death protein 1; PD-L1, programed death-ligand 1; T-VEC, talimogene laherparepvec.

**Table 3:** Samples of FDA-approved monoclonal antibodies and their mechanisms of action [140, 141, 143, 147, 148].

Monoclonal antibodies	Mechanism of action/target	Type of cancer
Blinatumomab	CD19/CD3 bispecific T cell engager	ALL
Atezolizumab	PD-L1 inhibition	Baldder, NSCL, breast cancer
Avelumab	PD-L1 inhibition	Urothelial carcinoma, mark cell carcinoma
Bevacizumab	VEGF Inhibition	Colorectal, NSCLC, renal, glioblastoma, ovarian
Cemiplimab	PD-1 inhibiton	Cutaneous squamous cell carcinoma
Cetuximab	EFGR inhibition	Colorrectal
Daratumumab	Immune modulation of CD38	Multiple myeloma
Dinutuximab	GD-2 inhibition	Neuroblastoma
Durvalumab	PD-L1 inhibition	Lung cancer
Elotuzumab	NK cell activation	Multiple myeloma
Ipilimumab	CTLA-4 Inhibition	Multiple myeloma, renal cell carcinoma
Isatuximab	CD38 inhibiton	Multiple myeloma
Mogamulizumab	CCR4 inhibition	Cutanoeus T cell lymphoma
Necitumumab	EFGR inhibition	NSCLC
Nivolumab	PD-1 inhibiton	Meloma, lung, renal
Obinutuzumab	CD20 inhibition	CLL
Ofatumumab	CD20 inhibition	CLL
Olaratumab	PDGFR*α* inhibition	Sarcoma
Panitumumab	EFGR inhibition	Colorectal cancer
Pembrolizumab	Inhibition of PD-1/PD-L1 signaling	Melanoma
Pertuzumab	HER2 inhibition	Breast cancer
Ramucirumab	VEGF-R2 inhibition	Gastric cancer
Transtuzumab	HER2 inhibition	Breast cancer
Rituximab	CD20 inhibition	NHL
Alemtuzumab	CD52 inhibition	CLL
Brentuximab	CD30 inhibition	Hodgkin lymphoma
Denosumab	RANKL inhibition	Cancer-induced bone destruction
Gemtuzumab	CD33 inhibition	Acute myeloid leukemia
Inotuzumab	CD22 inhibition	ALL
Polatuzumab	CD79B inhibition	B cell lymphoma
Fresolimumab	TGF-*β* inhibition	Breast cancer
Ibritumomab	CD20 inhibition	B cell NHL
Ocrelizumab	CD20 inhibition	Breast cancer
Mosunetuzumab	CD20 inhibition	B cell NHL
Plerixafor	Inhibits CXCR4 receptor on hematopoietic stem cell	NHL and multiple myeloma
Rituximab	CD20 inhibition	NHL, CLL
Brentuximab vedotin	CD30	Hodgkin lymphoma, ALCL
Margetuximab	HER2	HER2-positive breast cancer
Sacituzumab govitecan	Trop-2	TNBC
Enfortumab vedotin	Enfortumab vedotin	Urothelial carcinoma
Tisotumab vedotin	TF	Cervical cancer
Naxitamab	GD2	High-risk neuroblastoma
Loncastuximab tesirine	CD19	DLBCL
Belantamab mafodotin	BCMA	Multiple myeloma

Abbreviations: ALCL, anaplastic large cell lymphoma; ALL, acute lymphoblastic leukemia; BCMA, B-cell maturation antigen; CCR4, C–C chemokine receptor type 4; CLL, chronic lymphocytic leukemia; CTLA-4, cytotoxic T lymphocyte-associated protein 4; CXCR4, C–X–C chemokine receptor type 4; DLBCL, diffuse large B cell lymphoma; EFGR, epidermal growth factor receptor; GD2, disialoganglioside 2; GD-2, ganglioside GD2; HER2, human epidermal growth factor receptor 2; NHL, non-Hodgkin's Lymphoma; NK, natural killer; NSCLC, non-small cell lung cancer; PD-1, programed cell death protein 1; PD-L1, programed death-ligand 1; PDGFR*α*, platelet-derived growth factor receptor alpha; RANKL, receptor activator of nuclear factor kappa beta ligand; TF, tissue factor; TGF-*β*, transforming growth factor-beta; TNBC, triple-negative breast cancer; Trop-2, trophoblast cell surface antigen 2; VEGF, vascular endothelial growth factor; VEGF-R2, vasscular endothelial growth factor receptor 2.

## Data Availability

The data supporting the conclusions of this paper are available through the articles cited in the reference list.
